# Ski drives an acute increase in MMP‐9 gene expression and release in primary cardiac myofibroblasts

**DOI:** 10.14814/phy2.13897

**Published:** 2018-11-22

**Authors:** Natalie Landry, Morvarid S. Kavosh, Krista L. Filomeno, Sunil G. Rattan, Michael P. Czubryt, Ian M. C. Dixon

**Affiliations:** ^1^ Department of Physiology and Pathophysiology Institute of Cardiovascular Sciences Rady Faculty of Health Sciences Max Rady College of Medicine University of Manitoba Winnipeg Manitoba Canada

**Keywords:** Cardiac fibroblast, cardiac fibrosis, cell migration, fibroblast activation, MMP9, myofibroblast, Ski

## Abstract

Many etiologies of heart disease are characterized by expansion and remodeling of the cardiac extracellular matrix (ECM or matrix) which results in cardiac fibrosis. Cardiac fibrosis is mediated in cardiac fibroblasts by TGF‐*β*_1_/R‐Smad2/3 signaling. Matrix component proteins are synthesized by activated resident cardiac fibroblasts known as myofibroblasts (MFB). These events are causal to heart failure with diastolic dysfunction and reduced cardiac filling. We have shown that exogenous Ski, a TGF‐*β*_1_/Smad repressor, localizes in the cellular nucleus and deactivates cardiac myofibroblasts. This deactivation is associated with reduction of myofibroblast marker protein expression in vitro, including alpha smooth muscle actin (*α*‐SMA) and extracellular domain‐A (ED‐A) fibronectin. We hypothesize that Ski also acutely regulates MMP expression in cardiac MFB. While acute Ski overexpression in cardiac MFB in vitro was not associated with any change in intracellular MMP‐9 protein expression versus LacZ‐treated controls,exogenous Ski caused elevated MMP‐9 mRNA expression and increased MMP‐9 protein secretion versus controls. Zymographic analysis revealed increased MMP‐9‐specific gelatinase activity in myofibroblasts overexpressing Ski versus controls. Moreover, Ski expression was attended by reduced paxillin and focal adhesion kinase phosphorylation (FAK ‐ Tyr 397) versus controls. As myofibroblasts are hypersecretory and less motile relative to fibroblasts, Ski's reduction of paxillin and FAK expression may reflect the relative deactivation of myofibroblasts. Thus, in addition to its known antifibrotic effects, Ski overexpression elevates expression and extracellular secretion/release of MMP‐9 and thus may facilitate internal cytoskeletal remodeling as well as extracellular ECM components. Further, as acute TGF‐*β*_1_ treatment of primary cardiac MFB is known to cause rapid translocation of Ski to the nucleus, our data support an autoregulatory role for Ski in mediating cardiac ECM accumulation.

## Introduction

The cardiac extracellular matrix (ECM) is a load‐bearing connective tissue that is dysregulated in most etiologies of heart disease. The excessive deposition of ECM component proteins, such as fibrillar collagen, is manifested as cardiac fibrosis and ultimately contributes to heart failure (Martin and Blaxall 2012; Garza et al. 2015; Travers et al. 2016). The “cytokine hypothesis” upholds the contributions of many factors by intercellular cross‐talk among various cell types in the heart (Seta et al. 1996). TGF‐*β*_1_ and canonical downstream Smad2/3 signaling has been established as a major positive stimulus for cardiac fibrosis in heart (Hao et al. 1999; Wang et al. 2002, 2007; Biernacka et al. 2011) wherein resident cardiac fibroblasts (CF) lineages are implicated as contributing cells (Khalil et al. 2017), perhaps in combination with contributions of circulating fibrocytes and endothelial cell conversion by EndMT (Piera‐Velazquez et al. 2016). Resident CF are present in relatively large numbers within the myocardium and are known contributors to the synthesis and maintenance of the cardiac ECM (Eghbali 1992; Espira and Czubryt 2009). In many cardiac disease etiologies, the phenoconversion of CF to hyper‐synthetic cardiac myofibroblasts (MFB) precedes the inappropriate accumulation of fibrotic material and this increases global cardiac stiffness which may lead to decreased cardiac output and eventual heart failure (Weber et al. 2013).

Regulatory signaling for CF function by factors that influence ECM degradation and production, as well as cellular proliferation, migration, and phenotype conversion have become the focus for development of novel and effective antifibrotic treatments (Leask 2010). In this context, R‐Smad activation by TGF‐*β*_1_ is a key driver of myofibroblast‐mediated cardiac fibrosis and activation of CF (Leask 2010). Ski is a known repressor of the TGF‐*β*_1_ signaling pathway that disrupts R‐Smad/Smad4 complex signaling (Tecalco‐Cruz et al. 2018) in various cell types (Akiyoshi et al. 1999; Prunier et al. 2003; Ueki and Hayman 2003; Ferrand et al. 2010). We have noted that Ski rapidly translocates from the cytosol to the nucleus after treatment of MFB with TGF‐*β*_1_ (Cunnington et al. 2011). Overexpression of Ski in MFB induces rapid phenotypic and functional changes including significantly diminished synthesis and secretion of type I collagen, decreased contractility, and overall negative regulation of the myofibroblast phenotype, as indicated by decreased levels marker expression of *α*‐SMA and the ED‐A splice variant of fibronectin (Santiago et al. 2010; Cunnington et al., 2011). Reduction of ED‐A fibronectin is likely a means of reducing stored latent TGF‐*β*_1_as it has been shown to recruit latent TGF‐*β*‐binding protein‐1 (LTBP‐1) (Klingberg et al. 2018). Results from our recent studies indicate that Ski's antifibrotic actions may occur via the regulation of Zeb2 and Meox2 (Cunnington et al. 2014). Understanding the effects of Ski on other cellular processes such as MMP activation will provide additional information as to the efficacy of Ski‐based treatment approaches as putative antifibrotic therapy in cardiac fibrosis.

Normal cardiac ECM function is based on the balance between accumulation and removal of the ECM that maintains myocardial structure (Goldsmith et al. 2013; Tao et al. 2014; Ueland et al. 2015). ECM homeostasis is primarily determined by the release profiles of hydrolytic proteases, such as matrix metalloproteinases (MMPs) (Fan et al. 2012; Moore et al. 2012; Zhang et al. 2017). MMP‐9 (and MMP‐2) are the most abundant proteases in cardiac tissue, and MMP‐9 is a key modulator of the postinjury ECM remodeling in heart (Jacob‐Ferreira and Schulz 2013; Iyer et al. 2016; Lindsey 2018). Secretion of MMPs appears to be regulated by external signals as well as cellular differentiation (Welgus et al. 1990), while their activation is influenced by release of growth factors and cytokines from their degraded substrate (Clutterbuck et al. 2009). In the adult heart, MMP‐9 regulates cardiac interstitial matrix homeostasis (Iyer et al. 2016) due to its specific affinity for various collagens (Lauer et al. 2014), as well as fibronectin, laminin, tenascin‐B, and thrombospondin (Iyer et al. 2016) and is robustly elevated in multiple cardiovascular diseases (Iyer et al. 2016). Attenuation of left ventricular remodeling has been observed in MMP‐9 knock‐out transgenic mice (Mukherjee et al. 2006). Cardiac matrix remodeling and altered MMP activity in response to cardiac injury are also associated with changes in cardiac cell adhesion to the matrix itself (Fan et al. 2012, 2014; Moore et al. 2012), which is mediated by proteins such as focal adhesion kinase (FAK) and integrins, the primary component of cell‐matrix adhesion complexes.

As Ski is a known antifibrotic factor in cardiac MFB (Cunnington et al., 2011) we hypothesized that Ski overexpression in MFB will regulate expression of MMP‐9 and thus may influence MMP‐9 protease release in vitro. This in turn may reflect a novel pathway for the regulation of cardiac ECM remodeling.

## Materials and Methods

### Cell culture and adenoviral infection

All animal studies protocols were approved by the Animal Care Committee of the University of Manitoba in accordance with the Canadian Council on Animal Care guidelines. Cardiac fibroblasts were isolated from adult male Sprague Dawley rats and passaged to P1 MFB as previously described (Ju et al. 1998, Cunnington et al., 2011; Cunnington et al. 2014). For adenoviral treatments, cells were grown to 70% confluency and infected for 48 h with either an HA‐tagged Ski‐expressing adenoviral vector (AdSki), described in a previous study (Cunnington et al., 2011) or an LacZ‐expressing adenovirus (AdLacZ) at varying multiplicities of infection (MOI) in serum‐free DMEM/F12 (1:1) media supplemented with 100 units/mL penicillin‐streptomycin and 100 *μ*mol/L ascorbic acid. Cells were harvested by scraping in RIPA lysis buffer, and total protein concentration was determined using a bicinchoninic acid colorimetric assay.

### Western blotting

For whole‐cell lysates, equal amounts (10–20 *μ*g) of protein were loaded onto 8–12% SDS‐polyacrylamide gels for separation, followed by transfer to polyvinylidene difluoride (PVDF) membrane. When probing for secreted proteins, equal volumes of concentrated, conditioned media were loaded (30‐40 *μ*L/well). Membranes were blocked with 10% nonfat milk in phosphate‐buffered saline (PBS) for 1 h at room temperature on an orbital shaker. Following several washes in PBS, the membranes were incubated in primary antibody, diluted according to the manufacturers’ recommendations in PBS with 3% nonfat milk for either 90 min at room temperature or overnight at 4°C. Primary antibodies used included: Anti‐Ski (Upstate/Millipore), anti‐HA epitope tag (Rockland Immunochemicals), anti‐*β*‐tubulin, anti‐MMP‐2 C‐terminal (ab79781, abcam), anti‐MMP‐9 (EP1254), anti‐integrin‐*β*1 (EPR16895), antipaxillin (5H11, DSHB), antivinculin (Millipore), anti‐TIMP‐3 (Chemicon), anti‐TIMP‐4 (Chemicon), anti‐FAK (3285, Cell Signaling Technology), and anti‐pFAK (Tyr397, Biosource). Membranes were then washed several times in PBS with 0.2% Tween‐20 (PBS‐T), then incubated at room temperature with secondary horseradish peroxidase (HRP) conjugated goat anti‐mouse or anti‐rabbit antibodies (1:10,000; Jackson Laboratories, Bar Harbor, ME) in PBS‐T with 3% milk for 1 h. Protein bands were then visualized using Pierce ECL western Blotting Substrate or SuperSignal West Pico Chemiluminescent Substrate (Thermo Fisher Scientific, Canada) then exposing to CL‐XPosure film (Thermo Scientific, Canada). Densitometric analysis was conducted using Quantity One software (version 4.6.9; Bio‐Rad Laboratories, Hercules, CA, USA). For cellular protein lysate samples, observed protein levels were normalized to *β*‐tubulin whereas samples from conditioned media containing MMP target secreted proteins were normalized to untreated controls, as previously described (Hittel et al. 2009; Ghavami et al. 2014).

### RNA isolation and quantitative PCR

Control and AdSki‐infected primary cardiac fibroblasts were isolated by trypsinization and centrifugation at 200*g* for 5 min. Column‐based RNA isolation from cell pellets was performed using the PureLink RNA Mini kit (Invitrogen, Carlsbad, CA) according to the manufacturer's instructions. Two‐step qPCR was performed first by treating the isolated RNA with DNase I (New England Biolabs) and synthesizing cDNA from 100 ng of RNA, using the SuperScript VILO Master Mix (Invitrogen). Reactions were prepared using 1 *μ*L of cDNA template and 200 nM of forward and reverse primers in a final volume of 10 *μ*L. PCR amplification was performed using PowerUp SYBR Green Master Mix (Applied Biosystems, Foster City, CA), in triplicate for each reaction on a QuantStudio 3 Real‐Time PCR System (Applied Biosystems). The following cycling program was used: UDG activation at 50°C (2 min), then 95°C (2 min), followed by 40 cycles of denaturation at 95°C (15 sec), and extension at 60°C (30 sec). After amplification, a continuous melt curve was generated from 60 to 95°C. Relative gene expression was calculated using the 2^−ΔΔCt^ method (Livak and Schmittgen 2001), using untreated cells as controls, and normalized to *Gapdh*. Rat‐specific primer pairs used were: *Gapdh* forward 5′‐TGCACCACCAACTGCTTAGC‐3′ and reverse 5′‐ACGGCCATCACGCCACAGC‐3′; *Mmp2* forward 5′‐ACCGTCGCCCATCATCAA‐3′ and reverse 5′‐TTGCACTGCCAACTCTTTGTCT‐3′; *Mmp9* forward 5′‐TCGAAGGCGACCTCAAGTG‐3′ and reverse 5′‐TTCGGTGTAGCTTTGGATCCA‐3′.

### Gelatin zymography

Samples containing 10–15 *μ*g of total protein were loaded onto either Novex 10% Zymogram Protein Gels (Life Technologies, Burlington, ON Canada) or gelatin substrate acrylamide gels and separated at a constant voltage. Gels were incubated in developing buffer (500 mmol/L Tris‐HCl, pH 7.8, 2 mol/L NaCl, 50 mmol/L CaCl_2_, and 0.2% v/v. Brij 35) and stained with Coomassie blue reagent (0.5% w/v Coomassie blue R‐250, 5% v/v. methanol, and 10% v/v acetic acid), followed by destaining with 10% methanol/5% acetic acid solution until areas of gelatinolytic activity were observable as clear bands. Dried gels were imaged using Image J software (National Institutes of Health, Bethesda, MD, USA) (Schneider et al. 2012).

### Statistical analysis

The values for all data correspond to mean ± standard error of means (SEM). The histographical representation of densitometric analysis was provided wherein each treatment group was normalized to the control value of that experiment and then averages of each experiment was shown relative to control. All experiments were repeated with a minimum of 3 biological replicates (*n* = 3) with primary cells isolated from three different animals and cultured separately to assemble the statistical cohort (see Figure legends). For all experimental data with multiple samples, the statistical significance of differences between means was determined by repeated measures one‐way analysis of variance (ANOVA) with a Brown–Forsythe test for equal variance and Tukey posthoc analysis. Differences between groups were considered to be statistically significant when *P* ≤ 0.05.

## Results

### Effects of exogenous Ski expression on MMP expression and activity in cardiac MFB

**Figure 1:**
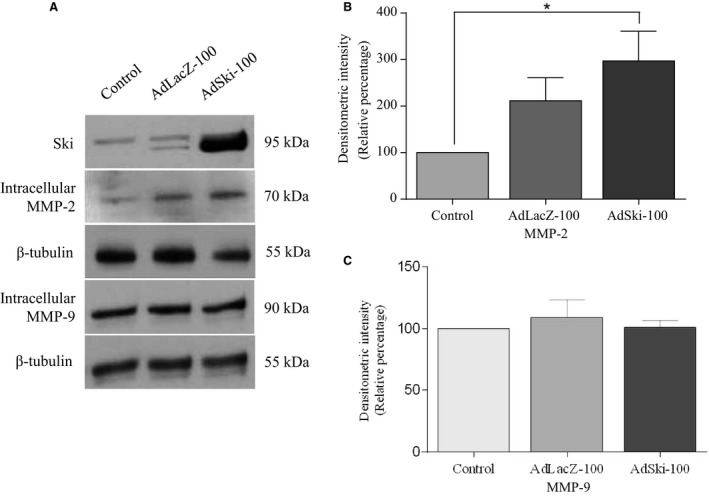
Ski overexpression increases intracellular MMP2 expression in primary cardiac myofibroblasts. First passage (P1) cardiac MFB grown to 70% confluency, and infected with either Ad‐LacZ (LacZ‐100) or AdSki (Ski‐100) at MOI of 100 for 48 h. Untreated control cells were serum‐deprived, but not infected with either virus. (A) Representative immunoblots of total cell lysates probed for intracellular Ski, MMP‐2, *β*‐tubulin (control), and MMP‐9 with *β*‐tubulin (control). (B) Histogram showing data obtained by densitometry of blots as in A. The level of intracellular MMP‐2 was significantly increased in response to Ski overexpression, compared to untreated controls. Data are representative of the means ± SEM with *n* = 4 replicates. *P < 0.05 compared to uninfected controls. Untreated control lysates were serum‐starved, but not infected with either virus. (C) Histogram showing data obtained from Western blots of total cell lysates probed for intracellular MMP‐9. Data are normalized to *β*‐tubulin +/‐ SEM **.** The levels of intracellular MMP‐9 was not altered in response to Ski overexpression (AdSki‐100). Data are representative of the means ± SEM;* n* = 8; *P* = 0.7394.

To investigate the effects of adenoviral Ski over‐expression on intracellular cardiac P1 MFB MMP‐2 and MMP‐9 expression, respectively, we infected cells with Ski‐expressing adenovirus or LacZ‐expressing control adenovirus at 100 MOI for 48 h (Fig. 1A).

### MMP‐2

**Figure 2:**
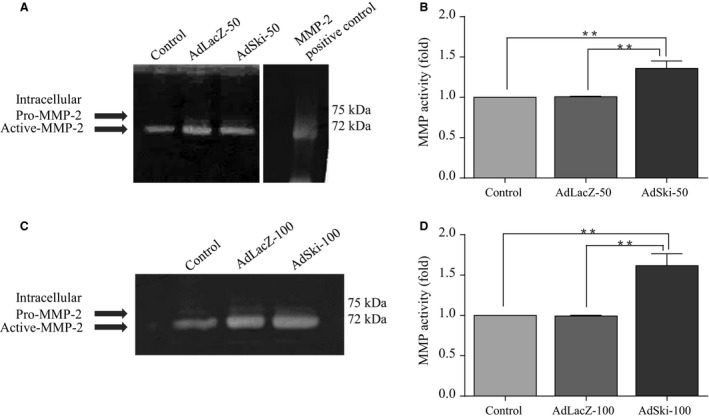
Ectopic Ski expression increases MMP2 gelatinase activity in primary cardiac myofibroblasts. First passage (P1) primary cardiac MFB were grown to 70% confluency, and infected with either AdLacZ or AdSki at 50 and 100 MOI for 48 h. Untreated control lysates were serum‐deprived, but not infected with either virus. (A and C) Representative gelatin zymographs of total cell lysates for intracellular MMP‐2 in MFB treated with 50 MOI adenovirus (Fig. 2A) or 100 MOI adenovirus (Fig. 2C). Native human MMP‐2 protein (5 *μ*g) was used as a positive control in Figure 2A. (B and D) Histograms showing data obtained in Figures 2A and C, respectively. The level of intracellular MMP‐2 activity was significantly increased in response to Ski overexpression, compared to Ad‐LacZ and untreated controls. Data are representative of the means ± SEM with *n* = 3. **P < 0.01, compared to uninfected and Ad‐LacZ infected controls.

**Figure 3:**
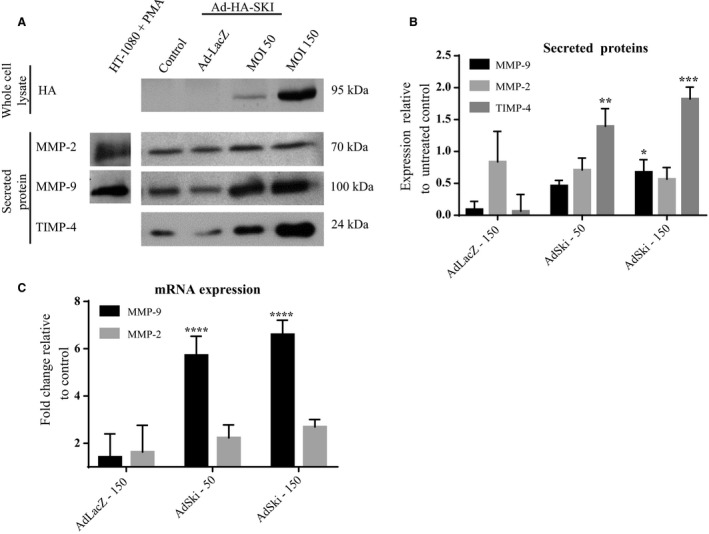
Ski overexpression upregulates MMP‐9 expression in primary cardiac myofibroblasts. First passage (P1) primary rat cardiac MFB were grown to 70% confluency, and infected with either Ad‐LacZ or Ad‐Ski at 50 and 150 MOI for 48 h. Untreated control MFB cultured cells were serum‐deprived, but not infected with either virus. Total cell lysates were assayed for Ski to confirm successful infection of cells. Conditioned media was assayed for MMP‐2 protein via immunoblotting. Data are normalized to the control expression levels ± SEM. (A) Representative immunoblots of concentrated conditioned media (20 *μ*g/lane) for secreted MMP‐2, MMP‐9, and TIMP‐4 as well as cell lysates for HA‐tagged Ski. Specificity of bands for MMP‐2 and MMP‐9 were noted with the use of HT‐1080 cells treated with PMA and assayed from conditioned media using the same Western blot conditions as provided to our lab (Richard Schulz, University of Alberta, personal communication). (B) Histogram showing data obtained from Western blots, as represented in Figure 3A. The level of secreted MMP‐2 was unchanged in response to AdSki infection. Data are representative of means ± SEM, where *n* = 3. The level of secreted MMP‐9 was significantly increased in AdSki‐infected cells compared to Ad‐LacZ‐infected controls. Data are representative of the means ± SEM where *n* = 7; *P ≤ 0.05 compared to untreated and LacZ controls. Protein levels of secreted TIMP‐4 were significantly reduced with AdSki infection, compared to AdLacZ‐infected and uninfected controls. Data are representative of the means ± SEM where *n* = 7; **P ≤ 0.01 and ***P ≤ 0.001, when comparing to untreated and AdLacZ‐50 and AdLacZ‐150 controls, respectively. (C) qPCR assay for expression of MMP‐2 and MMP‐9 mRNA expression in AdSki treated samples versus controls. The histogram shows data normalized to *Gapdh*, ±SEM, where *n* = 3, ****P ≤ 0.001 using one‐way ANOVA, relative to untreated and AdLacZ controls.

**Figure 4:**
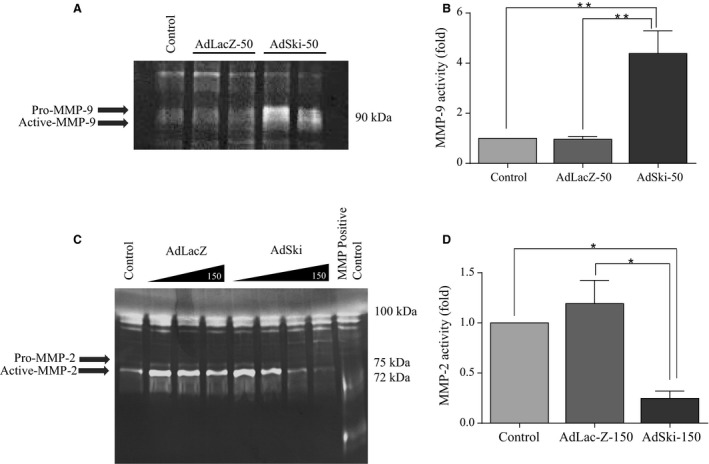
Ectopic expression of Ski yields opposite effects on MMP‐2 and MMP‐9 gelatinase activity in cardiac myofibroblasts. First passage (P1) primary cardiac MFB were grown to 70% confluency, serum‐deprived and infected with either Ad‐LacZ or Ad‐Ski at 50 MOI (for the MMP‐9 assay) or a range of 10 to 150 MOI (for the MMP‐2 assay) for 48 h. Untreated controls were serum‐deprived, but not infected with either virus. Concentrated conditioned media (15 *μ*g protein/lane) from all groups were assayed for MMP activity by gelatin zymography. Native human MMP‐2 protein (5 *μ*g) served as a positive control in MMP‐2 studies. (A) Representative gelatin zymography of concentrated conditioned media for secreted MMP‐9. (B) Histogram showing data obtained in A. Data are normalized to the control expression ± SEM. The level of secreted MMP‐9 activity was significantly elevated in response to Ski overexpression at an AdSki MOI of 50. Data are representative of the mean ± SEM where *n* = 3; **P ≤ 0.01 compared to untreated control and AdLacZ‐50 control values. (C) Representative gelatin zymography of concentrated conditioned media (15 *μ*g protein/lane) from all groups for MMP‐2 activity. The level of secreted MMP‐2 activity was significantly attenuated in response to Ski overexpression at MOI of 150, relative to LacZ‐infected controls. (D) Histogram showing data obtained in Figure 4C. Data are normalized to the control expression level ± SEM. MMP‐2 activity is significantly decreased in response to AdSki treatment compared to AdLacZ treated and uninfected controls. Data are representative of the means ± SEM where *n* = 3. *P ≤ 0.05 compared to untreated and AdLacZ‐150 controls.

Western analysis and data treatment of media samples for secreted proteins were carried out as noted previously (Ghavami et al. 2014). Western blotting revealed a significant increase in intracellular protein levels of MMP‐2 for cells infected with AdSki, versus control AdLacZ‐infected cells (Fig. 1B). Gelatin zymography indicated increased intracellular activity of MMP‐2 in AdSki‐infected cells (Fig. 2A–D). On the other hand, infection of cardiac myofibroblasts with AdSki was associated with no effect on secretion of MMP‐2 protein nor any change in MMP‐2 mRNAs in MFB (Fig. 3A–C). A gelatin zymogram (Fig. 4C and D) of conditioned media indicates significant reduction of MMP‐2 gelatinase activity in the media in the presence of exogenous Ski expression.

### MMP‐9

**Figure 5:**
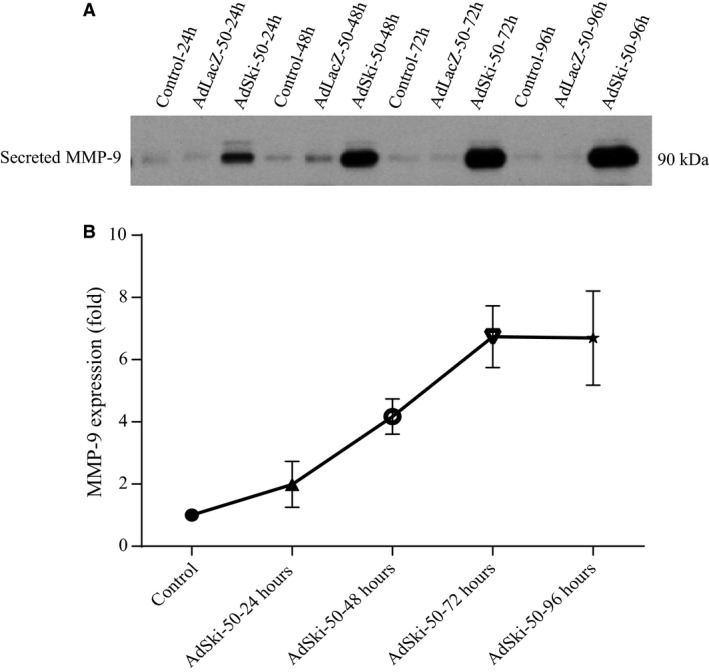
Chronic overexpression of Ski yields and increase in MMP‐9 secretion over time. P1 cardiac MFB were grown to 70% confluency and were infected with either Ad‐LacZ (MOI of 50) or AdSki (MOI of 50) for various durations up to 96 h. Untreated controls were serum‐deprived, but not infected with either virus. Expression of secreted MMP‐9 was assessed at 24, 48, 72, and 96 h after infection with AdSki. Using mean ± SEM where *n* = 3, we observed an significant increase in MMP‐9 expression over time, as seen at72 versus 48 h (*P ≤ 0.05) and 96 versus 24 h samples (*P ≤ 0.05).

Comparison of different treatment groups revealed that protein expression of intracellular MMP‐9 was not significantly different in MFB that were overexpressing Ski versus controls (Fig. 1A and C); this finding notwithstanding, we observed a significant increase in the level of secreted MMP‐9 protein when first‐passage (P1) MFB were infected with AdSki for 48 h at both 50 and 150 MOI, as compared to AdLacZ controls (Fig. 3A and B). Further, Ski overexpression is causal to elevated MMP‐9 mRNA abundance in transfected MFB's versus controls (Fig. 3C). The effect of Ski overexpression the secretion of MMP‐9 was time‐dependent (AdSki at 50 MOI), increasing significantly at 48, 72, and 96 versus 24 h (AdSki ‐ 50 MOI), with a peak in expression at 72 h after the induction of exogenous Ski (Fig. 5A and B). Gelatin zymography of conditioned media at 48 h indicated a significant increase in MMP‐9 activity in AdSki‐infected cells, compared to AdLacZ‐infected controls (Fig. 4A and B).

### Effects of Ski overexpression on TIMP‐4 in cardiac MFB

We also sought to determine the effects of Ski over‐expression on TIMP levels in P1 cardiac MFB, and thus examined secreted TIMP4 in P1 MFB with 50 and 150 MOI AdSki treatments. In response to AdSki infection (50 and 150 MOI for 48 h) we observed a significant increase in levels of secreted TIMP4 (24 kDa) compared to AdLacZ‐infected controls (Fig. 3A and B).

### The influence of Ski on MFB expression of focal adhesion proteins

**Figure 6:**
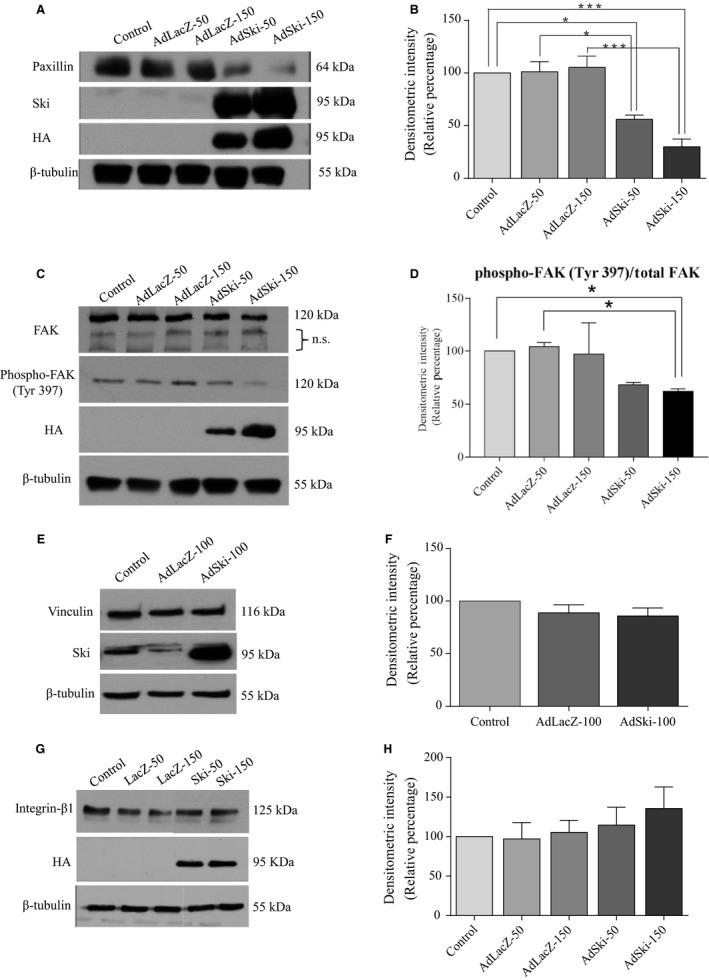
Paxilin expression and FAK phosphorylation are negatively regulated by Ski in cardiac myofibroblasts. First passage (P1) cardiac MFB were grown to reach 70% confluency and infected with either Ad‐LacZ or AdSki (MOI of 50, 100, or 150) for 48 h. Untreated controls were serum‐deprived, but not infected with either virus. Expression of exogenous HA‐tagged Ski was confirmed using anti‐HA antibodies and *β*‐tubulin was used as a loading control. (A) Representative immunoblots of total cell lysates probed for paxillin. (B) Histogram showing data obtained in A. Paxillin protein levels are significantly decreased in response to AdSki infection, as compared to AdLacZ‐infected and uninfected controls. Data shown are representative of the means ± SEM where *n* = 3; *P ≤ 0.05 for AdSki50 compared to untreated and AdLacZ‐50 controls; ***P ≤ 0.001 for AdSki150 compared to untreated and AdLacZ‐150 controls. **C:** Representative immunoblots of whole cell lysates probed for immunoreactivity to total FAK and phospho‐FAK (Tyr 397). n.s., non‐specific antibody binding. (D) Histogram showing data obtained in A. FAK phosphorylation at Tyr 397 is significantly decreased with Ad‐Ski infection at 150 MOI, compared to AdLacZ‐50 and uninfected controls. Data are representative of the mean ± SEM where *n* = 4; *P ≤ 0.05 for AdSki150 compared to controls. (E) Representative immunoblots of total cell lysates probed with vinculin antibody. (F) Histogram showing data obtained in E. No significant differences were observed among treatment groups. Data shown are representative of the means mean ± SEM where *n* = 8; *P* = 0.2619. (G) Representative immunoblots of total cell lysates for integrin‐*β*1 antibody. (H) Histogram showing data obtained in A. Data shown are representative of the mean ± SEM where *n* = 4; *P* < 0.6506.

In addition to organization of *α*SMA to internal stress fibres and expression of ED‐A fibronectin which enhances recruitment of latent TGF*β*_1_ binding protein‐1 (LTBP‐1) to the fibroblasts’ matrix (Klingberg et al. 2018),a key feature of the myofibroblast phenotype is expression of focal adhesion‐related proteins, such as paxillin (Santiago et al. 2010). Paxillin is a component of focal adhesions that provides a scaffold for recruitment of other focal adhesion components. In response to infection with AdSki (at either 50 MOI or150 MOI for 48 h), paxillin protein levels were significantly decreased as assessedby Western blotting, compared to Ad‐LacZ‐infected control MFB (Fig. 6A and B). Also, focal adhesion kinase (FAK) is a critical protein involved in formation and turnover of focal adhesions in MFB. Phosphorylation of FAK (Tyr 397) is indicative of increased FAK activity (Parsons 2003). Similar to Ski's effect on paxillin protein expression, Western blot analysis revealed that Tyr 397 phosphorylation of FAK was significantly decreased in response to over‐expression of Ski (AdSki at 150 MOI for 48 h) compared to uninfected controls (Fig. 6C and D). These effects were specific, as Ski overexpression (AdSki, 100 MOI, 48 h) did not induce significant changes in levels of vinculin, a focal adhesion protein that links focal adhesions to the actin cytoskeleton (Fig. 6E and F). Similarly, protein expression of integrin‐*β*1, a membrane‐spanning protein which links focal adhesions to the cell exterior was unchanged in response to increasing levels of AdSki infection (50 and 150 MOI for 48 h) (Fig. 6G and H).

## Discussion

### Ski is a modulator of MMP‐9 expression and MMP‐2 activity

The relative production and accumulation of ECM in cardiovascular tissues may represent a balance among production of matrix components and their removal, and that the latter may depend upon the release of hydrolytic proteases including MMP‐9 (Zhang et al. 2017), which is the most‐studied MMP in cardiac remodeling (Lindsey 2018). In the current study, we show that Ski overexpression is associated with increased MMP‐9 mRNA expression and protein secretion. In contrast, we noted no change in the release of MMP‐2 from MFB coupled with reduced MMP‐2 activity in the media of MFB cultures in the presence of Ski overexpression. These findings reflect the specificity of the effect of Ski, and indicate a potential role for Ski in regulating the secretion/release of MMPs from these cells. As MFB are important in maintaining homeostasis of the cardiac matrix, their control over the release and activation of hydrolytic protease MMPs may contribute to cardiac matrix remodeling in heart disease (Iyer et al. 2016). Hydrolytic proteases may exert a variety of effects on matrix, especially when considered in the acute setting (eg. fibroblast migration) or in chronic disease (remodeling of ECM). It is known that MMP‐9 processes an array of different protein substrates, including structural ECM proteins and latent TGF‐*β*_1_ (Sternlicht and Werb 2001), and the chronic overexpression of MMP‐9 in macrophages exacerbate age‐dependent cardiac dysfunction with attendant cardiac fibrosis (Toba et al. 2017). To the best of our knowledge, the current finding is the first report of regulation by Ski of MMP‐9 expression and release in primary cardiac myofibroblast cells.

Although we found that Ski overexpression significantly increased secretion of MMP‐9, intracellular MMP‐9 levels remained unchanged. This may be due to a compensatory mechanism existing in CF with respect to MMP levels, which requires further examination. We also found that TIMP‐4 expression was significantly elevated in the presence of Ski overexpression. Although the role of TIMP‐4 in CF and in cardiac fibrosis remains unclear, relatively high levels of expression of TIMP‐4 in control hearts are associated with preservation of normal matrix distribution when compared to reduced expression observed in human patients with overt cardiac fibrosis (Sakamuri et al. 2016). We speculate that the elevation of TIMP‐4 expression in our hands may be related to the inactivation of the MFB phenotype in these cells with exogenous Ski expression as we previously reported (Cunnington et al., 2011, Cunnington et al. 2014).

Ski's inhibitory effect on secreted MMP‐2 activity inhibition may be complex, and we suggest that Ski may play a transient regulatory role in the matrix remodeling process mediated by MMP‐2. Previous studies have shown that MMP‐2 activation is accompanied by increased type I collagen synthesis, for which FAK auto‐phosphorylation (Tyr 397) was required (Hori et al. 2012). As we observed a decrease in FAK phosphorylation in this residue with Ski overexpression, the additive nature of these effects supports a role for Ski in attenuation of the myofibroblast phenotype. This finding is supported by previous work from our lab demonstrating decreased collagen production with Ski overexpression (Cunnington et al., 2011). In addition, the apparent increase in the intracellular expression and activity of MMP‐2 with ectopic Ski expression may be an indicative of physiological changes within MFB. While MMP activation is canonically observed upon cleaving of the pro‐domain, MMP‐2 can also be activated by several post‐translational modifications, independent of pro‐peptide release (Schulz 2007; Decoux et al. 2014). Several subcellular elements are known to be affected by MMP‐2 proteolytic activity, including mitochondria, the cytoskeleton, as well as Calponin‐1 and Troponin‐I in cardiomyocytes (Castro et al. 2012; Hughes et al. 2014). Inhibition of intracellular MMP‐2 in cardiomyocytes subjected to ischemia‐reperfusion injury showed improvements in contractile recovery, and this was entirely independent of its extracellular activity (Jacob‐Ferreira and Schulz 2013). Furthermore, MMP‐2 has been shown to co‐localize with *α*‐actinin in cardiomyocytes, leading to a decrease in the formation of actin–myosin bridges and cell contractility (Schulz 2007). In the context of cardiac MFB, the actin cytoskeleton plays an integral role in the formation of stress fibres, lending a contractile phenotype to chronically activated MFB. While intracellular MMP‐2 activity has not been extensively studied in cardiac MFB, the increase in MMP‐2 activity observed with Ski overexpression may be an indicative of a beneficial change in cytoskeletal dynamics, leading to a less contractile, more fibroblast‐like phenotype. The physiological role of intracellular MMP‐2 activity in cardiac fibroblasts remains elusive; however it would be a novel player in the current understanding of the MFB phenotype seen in chronic wound healing. Nevertheless, the role of MMP‐2 in the activation of cardiac MFB requires further examination.

### Ski expression and reduced expression of paxillin and FAK (Tyr 397)

We observed a down‐regulation of the focal adhesion protein paxillin, as well as FAK (Tyr 397) phosphorylation in the presence of exogenous Ski. This effect appears to be highly specific, as Ski overexpression did not affect phosphorylation of Pyk2 (data not shown), another kinase with structure highly similar to that of FAK (Mitra et al. 2005). The specificity of paxillin and phosphorylated FAK as targets for Ski is also strengthened by the lack of response of vinculin or integrin‐*β*1 to treatment. We suggest that as focal adhesion‐associated paxillin and FAK are involved in cellular adherence to matrix component proteins, and cardiac MFB treated with Ski may be less adherent to matrix.

### Ski as a negative regulator of cardiac extracellular matrix proteins: interplay of factors

**Figure 7:**
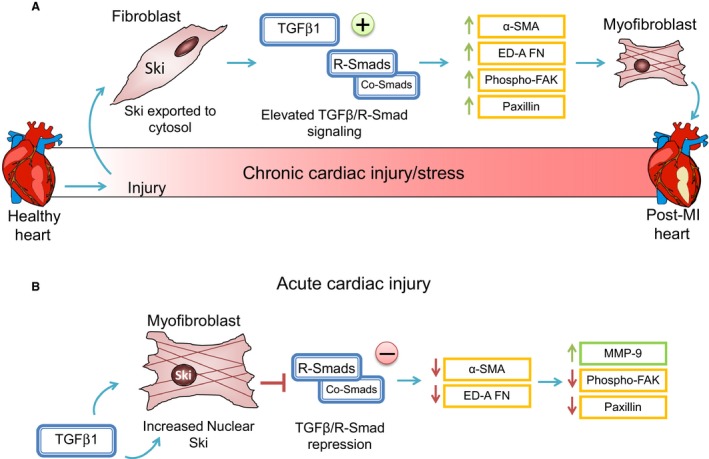
A schematic depicting Ski function in acute cardiac MFB activation. Herein we summarize our main findings, indicating that overexpression of Ski alone is sufficient to specifically and MMP‐9 secretion in MFB. We suggest that upon acute activation of the cell, Ski moves from the cytosol to the nucleus to repress TGF‐*β*_1_ and, as our current data indicates, serves as a driver of MMP‐9 expression and release. We hypothesize that Ski exerts its anti‐fibrotic effects, in part, by causing enhanced release of MMP‐9 in acutely stimulated cardiac fibroblasts and in cardiac myofibroblasts. To effect this specific response in combination with decreased MFB marker proteins and adhesion proteins (FAK and paxillin) Ski may serve as a scaffold for number of cofactors including Smads (Tecalco‐Cruz et al. 2018). On the other hand, in chronic cardiac injury (infarcted hearts) we have found that the intracellular distribution of Ski is shifted to the cytosol (Cunnington et al. 2011). If Ski cannot reach the nucleus, its effectiveness as a repressor of TGF‐*β*_1_ is compromised. Thus, in the chronically damaged and fibrosed heart, compartmentalization of Ski results in chronic elevation of MFB marker proteins and activation of the MFB phenotype.

Our current findings, in combination with our previous work (Cunnington et al. 2011, 2014) support multiple antifibrotic functions of Ski in cardiac MFB (see Fig. 7). While Ski effectively sequesters and binds Smads, which are needed for the canonical response to TGF‐*β*_1_, the mechanisms by which Ski modulates these functions may extend beyond its role as an endogenous repressor of TGF‐*β*_1_ (Tecalco‐Cruz et al. 2018). We have shown that TGF‐*β*_1_ itself leads to activation and movement of Ski to the nucleus (bound to R‐Smads) in MFB (Cunnington et al., 2011). Importantly, some evidence exists to indicate that Ski is a scaffolding protein that may bind to transcription factors such as c‐Jun (Pessah et al. 2002). Previous studies confirm that Ski binds to a variety of transcription factors and can act as both a co‐activator and co‐repressor, depending on its binding partners (Stavnezer et al. 1986; Nicol and Stavnezer 1998; Nomura et al. 1999) which may lend the fibroblast a modicum of flexibility with respect to a response to a given stimulus. Additionally, abnormal Ski compartmentalization as observed in MFB of the infarct scar of post‐MI hearts (Cunnington et al., 2011) may underpin the adverse remodeling and facilitate chronic cardiac fibrosis in the diseased myocardium (Fig. 7). Recently, we found that exogenous Ski inhibits the expression of Scleraxis, a pro‐fibrotic basic helix‐loop‐helix transcription factor, which has also been shown to regulate CF phenotype (Bagchi et al. 2016). As Scleraxis appears to inhibit MMP‐9 expression (Bagchi et al. 2016), its inhibition by Ski may serve a dual inhibitory role. In this regard, we have recently demonstrated that TGF*β*_1_‐mediated upregulation of MMP‐2 occurs by a Scleraxis‐dependent mechanism (Nagalingam et al. 2018), which again opposes the inhibitory effect of Ski on MMP‐2 activity in culture media as documented in the current dataset.

In the context of remodeling of the cardiac interstitium, it is suggested that Ski may serve to remove matrix proteins from the interstitial space, while Scleraxis may preserve matrix proteins from MMP‐9 based degradation, and that Ski and Scleraxis function in opposition. The accepted view of MMP‐9/TGF‐*β*_1_ activation is that of a bidirectional reciprocal regulatory loop, as the proteolytic cleavage of TGF‐*β*_1_ by MMP‐9 triggers TGF‐*β*_1_ function (Iyer et al. 2016). We suggest that Ski's influence on MMP‐9 adds a novel mode of control on cardiac matrix remodeling. In this context, MMP‐9 is a central player in matrix remodeling in heart disease including heart failure following myocardial infarction (Lindsey 2018) and recognizes common cardiac matrix substrates including collagens, fibronectin, laminin, thrombospondin, and tenascin‐C (Iyer et al. 2016). Thus while the importance of MMP‐9 in chronic heart failure is now established (Lindsey 2018), we propose that Ski may function as a pivotal regulator of MMP‐9 synthesis and release, particularly in the acute response of CF to stimuli that activate Ski and subsequent changes in cardiac matrix deposition and homeostasis of the cardiac ECM (Fig. 7). It is possible that the alteration of MMP‐9 release reflects an acute generalized effect of Ski, as MFB possess an extensive secretome that contributes to their role in wound healing and fibrosis. Given Ski's ability to attenuate the MFB phenotype, further assessment of whether the MFB secretome is grossly impacted by Ski over‐expression is warranted.

In summary, our data support the suggestion that Ski functions to drive proteolytic MMP‐9 gene expression and release from cardiac MFB. This effect occurs concomitantly with diminution of the MFB phenotype, as reflected in reduced expression of MFB marker proteins, as well as adhesion proteins FAK and paxillin. This effect may hasten removal of ECM proteins from the cardiac matrix, and thus may represent a novel mechanism for putative therapeutic modulation of myofibroblast function.

## Conflict of Interest

The authors declare that they have no competing interests.
